# Pregnant Women’s Perceptions of the Quality of Antenatal Care in a Public Hospital in Punjab, Pakistan during COVID-19: A Cross-Sectional Study

**DOI:** 10.3390/healthcare11070996

**Published:** 2023-03-31

**Authors:** Saima Tasneem, Macide Artac Ozdal

**Affiliations:** 1 Department of Health Management, Institute of Graduate Studies and Research, European University of Lefke, TRNC-10 Mersin, Lefke 99770, Northern Cyprus, Turkey; 2 Department of Health Management, Faculty of Health Sciences, European University of Lefke, TRNC-10 Mersin, Lefke 99770, Northern Cyprus, Turkey

**Keywords:** quality of antenatal care, maternal health, perinatal care, Pakistan, SDG 3, utilization of maternal health services, perceived quality of antenatal care, COVID-19

## Abstract

Despite government efforts, many rural Pakistani women forgo regular antenatal visits, are unprepared for birth, and deliver at home or in private facilities, because they are dissatisfied with public health services. This study examined pregnant women’s perceptions of public health hospital prenatal care to suggest areas for improvement. Using simple random sampling, 200 pregnant women visiting a secondary care public health facility in Sargodha District, Pakistan, were enrolled in a cross-sectional study. The quality of prenatal care was assessed using a structured and validated questionnaire. Descriptive analysis and multivariate linear regression stepwise models were used. Of participants, 52% consider the services to be of poor quality. Education, income, number of living children, and long waiting time influenced the perceived prenatal care quality in the study population. Stakeholders rated existing services as suboptimal, especially in terms of staff availability and time spent, which reduces service use. Facility managers and policymakers should work to improve the quality of services to satisfy patients, encourage them to use antenatal care, and improve the health of both mother and child, especially in rural areas.

## 1. Introduction

WHO data shows that 810 women die each day around the world due to pregnancy- and childbirth-related causes [1]. Alongside maternal mortality maternal morbidity, neonatal mortality, and stillbirths are other concerns for policymakers, especially in low- and middle-income countries (LMICs). Attaining Sustainable Development Goal 3, which encourages member nations to reduce maternal mortality to 70 per 1000 live births and newborn mortality to 12 per 1000 by the year 2030 [2], is a great challenge for low-resource countries such as Pakistan. Pakistan has a high maternal mortality ratio (186 per 100,000 live births) [3], a high neonatal death rate (300,000 newborn deaths annually) [4], and a high stillbirth rate (32 per thousand deliveries) when compared with other LMICs [3]. According to the current WHO guidelines, a pleasant pregnancy and birth experience is essential to person-centered care and is every childbearing woman’s right. The updated antenatal care (ANC) guideline by the WHO recommends that pregnant women have eight instead of four ANC visits during their pregnancy to improve pregnancy outcomes and gain better consumer satisfaction [4], but the latest available Pakistan Demographic and Health Survey Data shows that only 41.7% of women living in the rural areas of the country had four or more visits during their pregnancy [5].

In light of WHO recommendations on maternal health, various measures, such as free maternal and child health services at public health facilities and lady health worker programs, were adopted by the government of Pakistan to reduce perinatal mortality and complications. Despite these measures, the overall progress toward reducing MMR and NMR has been quite stagnant over the past couple of years [6]. Even before the onset of the pandemic, maternal and child health programs in the country faced challenges such as inadequate implementation of policies, administrative issues, human resource shortages, inconsistent supplies, and failure to provide high-quality maternal care [7]. Of expectant mothers, 50% in the rural areas of the country prefer deliveries at home by traditional birth attendants, because they believe that the quality of services offered at these public health facilities, the way they are treated by the healthcare providers, and the number and skills of staff at public facilities are not adequate [8]. This perceived poor quality results in delayed engagement or non-attendance during the antenatal period, poor birth preparedness, and complication readiness, and then leads to unwanted pregnancy outcomes [9].

The emergence of COVID-19 caused a further decline in the availability, accessibility, quality, and utilization of routine maternal and child health programs [10]. The redeployment of healthcare providers, reductions in staff numbers (because of sickness related to COVID-19), cancellations of appointments, and restrictions on community visits in the country (due to lockdowns) had a direct negative impact on the quality of care provided to pregnant and postpartum women and newborns [11]. In Pakistan, like other LMICs, healthcare personnel could not provide good care because there were many fears, restrictions, and precautions surrounding provider–user interactions [12]. The number of institutional deliveries and first postnatal visits decreased by 37% [13] when compared to the pre-COVID records.

Patients’ experiences with healthcare services are commonly acknowledged as a critical component of healthcare quality assurance and improvement, which holds true for the quality of maternal healthcare services [14]. Despite the fact that a few studies have been carried out to investigate how pregnant women perceive the quality of prenatal care in Pakistan [15,16,17,18], there is no available literature investigating pregnant women’s perceptions of the quality of prenatal care offered at the public health facilities in rural areas of Pakistan during the pandemic. This study aims at assessing how pregnant women living in the less developed areas of the country perceive the quality of prenatal care offered to them at the public health facility during the COVID-19 pandemic, and which of the sociodemographic, obstetric, or facility-related factors influence their evaluations. The results of the study will assist in identifying gaps that need attention to ensure the uninterrupted provision of quality care services in times of emergencies and pandemics in the future.

## 2. Materials and Methods

### 2.1. Study Design and Study Site

The country’s public healthcare system strives to provide healthcare through a three-tiered healthcare delivery system and a variety of public health activities. The first level of healthcare comprises Basic Health Units (BHUs) and Rural Health Centers (RHCs). This is the level of healthcare where patients have their first contact with the system and receive curative and preventative healthcare services [19]. Secondary care includes first- and second-referral facilities that provide acute, ambulatory, and inpatient care via the Tehsil Headquarter Hospitals (THQs) and District Headquarter Hospitals (DHQs). Tertiary care hospitals provide more specialized inpatient care, and these healthcare treatments are often reserved for inpatients and those referred by primary or secondary care physicians [20].

An institution-based cross-sectional quantitative descriptive study was carried out at a secondary care hospital in District Sargodha, Punjab, Pakistan. An exit interview strategy was employed. The research facility in this particular study serves a surrounding community of 52 different villages that benefit from the secondary level of medical care offered at the hospital. A network of ten basic health units and a team of 52 lady health workers are involved in providing these services to the community. 

### 2.2. Study Sample and Data Collection Methods

The total population for the current study comprised all the women of childbearing age who were pregnant at the time of the study with no pregnancy-related complication or chronic ailment, and who attended the antenatal clinic of the research facility and were planning to deliver in district Sargodha. Due to the country’s third wave of the pandemic and smart lockdowns implemented by local governments, the number of expectant mothers visiting outpatient departments was significantly low. During the first three months of 2021, on average, 330 patients visited the outpatient department for antenatal checkups. The sample size of 178 was determined using the online Raosoft sample size calculator, with a confidence level of 95% and a margin of error of 5% [21]. To avoid the problem of missing values and the nonresponse rate, 200 people were recruited using a simple random sampling technique. 

The pregnant women who visited the hospital in May and June 2021, irrespective of their gestational age, the number of visits, and past experiences, were requested to fill out the questionnaire to help assess their perceptions of the quality of the antenatal care services received during their current pregnancy.

A structured questionnaire comprising two sections was used for this purpose. Section A addressed the sociodemographic and maternal health characteristics of the participants. Section B was based on a pre-validated and structured instrument, the Quality of Prenatal Care Questionnaire (QPCQ), that indicates the woman’s perceptions of the quality of prenatal care received at the healthcare facility, used after seeking permission from the developer. The questionnaire assesses patient’s perspectives on quality based on six dimensions of care, namely information sharing, anticipatory guidance, sufficient time, approachability, availability, and support and respect. Section B comprised 46 items and used a Likert scale that ranked the responses from 1 (strongly disagree) to 5 (strongly agree) [22]. 

### 2.3. The Research Questions

The research questions were as follows:

- How do pregnant women perceive the overall quality of care they get at the particular health center, and is there a difference in the perceived quality of care in terms of the individual six dimensions studied?
- What is the influence of various socio-economic, pregnancy-related, and facility-related factors on the perceived quality of care?

### 2.4. Ethics Approval

The study methodology was approved by the European University of Lefke Ethics Committee with number ÜEK/57/01/12/2021/03 and the concerned authorities of the health facility with letter number 1074/MS. Informed consent was obtained from the study participants. All those who consented to participate requested to keep their credentials confidential.

### 2.5. Data Analysis

Statistical Package for Social Sciences (SPSS) version 25 was used for entering, cleaning, and analyzing the data. Descriptive analysis and tables were used to present the participants’ characteristics. For analyzing the women’s perceptions of the quality of antenatal care, the scoring scheme provided by the instrument developers was used. The instrument encompasses six aspects of quality of care that help reflect the women’s perceptions of the quality of care received during the course of their pregnancy, namely: information sharing (nine questions), anticipatory guidance (11 questions), sufficient time (five questions), approachability (four questions), availability (five questions), and support and respect (12 questions). Before the final analysis, questions 8, 15, 23, 28, and 40 were reverse-scored. The aggregate value of the QPCQ subscales is computed and reported as a total score, with a possible range of 46 to 230, with higher values suggesting a higher level of care. The total score is divided by 46, and the score of each subscale is divided by the corresponding number of questions that are included in that subscale. A higher value indicates higher-quality care. The mean score can be anywhere from 1 to 5, with 1 being the lowest and 5 being the highest quality of care. Moreover, to interpret participant QPCQ responses, the overall score was reported as a percentage of the theoretical maximum score (230), with scores above 70% suggesting good care and scores under 70% reflecting poor care [22].

Multivariate linear regression forward stepwise modeling was employed to identify the association between the perceived quality of prenatal care and various co-factors, such as education, income level, number of living children, number of antenatal visits, etc. Automatic linear modeling was done using SPSS, in which, after taking into account the marginal contribution of a predictor and accounting for other variables that are already in the model, a stepwise method approach adds or deletes predictors, one at a time. In forward stepwise, once each variable is added to the set of selected predictors, the algorithm checks if any of the previously selected variables might be eliminated without materially raising the residual sum of squares. This operates until the stop criteria are satisfied [23]. The variables that have outliers are transformed by using automatic linear regression model to achieve linearity and a better model fit [24]. The variables that were not significant were eliminated by using this technique, and the variables like income status, occupation of the husband, and realizing the significance of delivery by skilled birth attendants that had a significant impact were transformed and included in the final linear regression model. The adjusted R square value for the current model was 24.1%, which is acceptable in the human and social sciences [25].

## 3. Results

### 3.1. Sociodemographic Characteristics of Study Participants

The sociodemographic characteristics of study participants are given in Table 1. Of the women, 33.5% did not know their age; the mean age for the study participants was 29.32 years, with the minimum age being 18 years and the maximum age being 38 years; and 38.5% (77) of them had never received any formal education as they did not attend school. Of the mothers who participated in the study, 160 (80%) were residents of villages or small towns. Most of them were married by the age of 25. In the country, joint families are preferred over nuclear families, which was also the case with our study population, as 78% were living in a joint family unit. 

### 3.2. Pregnancy-Related Characteristics of Participants

Nearly 90% of the study participants were in their second and third trimesters of pregnancy. Though 49.5% were in the third trimester of pregnancy, just 4% had three antenatal visits. Of the women, 38% were primigravida. Detailed results for pregnancy-related characteristics are given in Table 2.

### 3.3. Hurdles in Accessing Available Healthcare Services, as Perceived by the Participants

Even though the research was conducted during the COVID-19 pandemic, only 5% of respondents considered the restrictions imposed by the government as a barrier to accessing healthcare services. The majority of the participants believed that their use of health services is influenced by factors, such as distance from the health facility, the duration of waiting time within the center, and their husbands’ negative attitudes towards antenatal care. Of the expectant mothers, 70.5% believed that pregnancy is not a health issue and 76.5% of participants failed to realize the significance of delivery in the presence of a skilled birth attendant. Details are given in Table 3.

### 3.4. Quality of Prenatal Care as Perceived by Mothers

**Figure 1:**
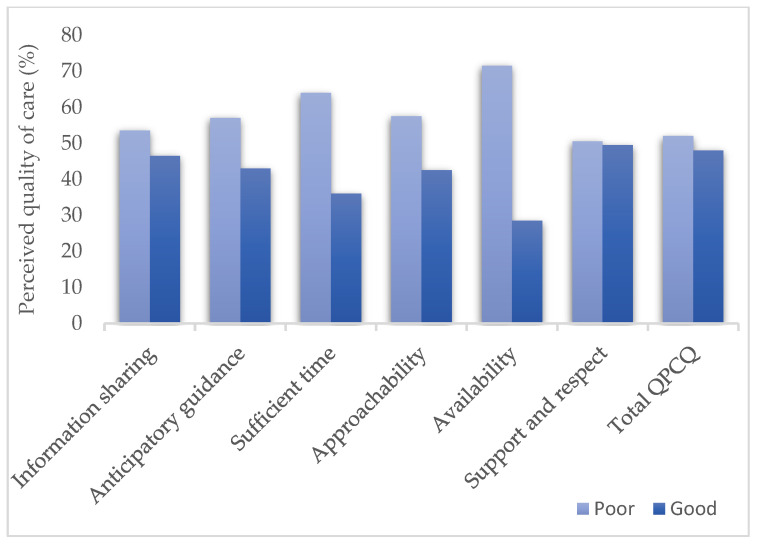
Quality of prenatal care as perceived by pregnant mothers.

Regarding the overall quality of prenatal care services, only 48% of participants considered the quality of services as good. Information sharing was considered inadequate by nearly 54% of the participants. Amongst the individual factors which comprised the quality instrument, the availability of skilled birth attendants scored the lowest (71.5% of participants) and 64% of women were not satisfied with the time that the healthcare provider spent with them (Figure 1).

### 3.5. Linear Regression Model for Factors That Influence Perceived Quality of Care

Forward stepwise linear regression model results for the predictor variables, after transformation, show that various variables, such as education level (*p* = 0.000), income (*p* = 0.009), number of living children (*p* = 0.033), and long waiting time (*p* = 0.049) have a significant influence on the perceived quality of care. Details are shown in Table 4.

## 4. Discussion

To the best of our knowledge, this is the only cross-sectional study that was done to assess the perceived quality of prenatal care provided to pregnant women at public healthcare facilities in rural areas of the country, and factors associated with their perceptions during the COVID-19 pandemic. 

In terms of perceived quality of care, this cross-sectional survey revealed that more than half of women (52%) believed that the antenatal services provided by the hospital were of poor quality, and only 48% viewed the services as being of good quality. Six variables were examined by QPCQ: information sharing, anticipatory guidance, sufficient time, approachability, availability, support, and respect. Though all six variables were graded as poor by the participants, the availability of healthcare workers in the facility was the biggest concern for the mothers who participated in the study, followed by sufficient time (71.5% and 64%, respectively). For the rest of the four variables, dissatisfaction was shown by more than 50% of the research participants. There are a variety of social factors beyond the control of the health sector that shape these perceptions, and these economic, social, and cultural variables influence service uptake and utilization [26]. The education status of pregnant mothers, their monthly family income, the number of living children that they have, and the time these women have to wait in the health facility were the final predictors of the total quality of prenatal care score in the study population.

In Pakistan, little attention has been paid to the patient’s experiences regarding the quality of prenatal care services provided at public health facilities. Despite all efforts, no research could be found aimed at assessing the quality of maternal health services offered in public health facilities, especially in the rural areas of Pakistan during the pandemic, focusing on patients’ perceptions based on their experiences.

When the results were compared with studies done before the COVID-19 pandemic in various LMICs, the perceived overall quality of maternal health services (52%) was lower than that in Tamil Nadu, India (98.5%) [27], Northern Ghana (81% and 85% for two districts) [28], Ethiopia (52.3%) [29], Bangladesh (54.8%, 97%) [30], and the Harari region, Ethiopia (70.3%) [31].This might be attributable to the tools used to collect data, differences in the perceptions of patients and their expectations, variations in their socioeconomic status, the availability of trained healthcare professionals, cultural differences, and the absence of COVID-19. The results were consistent with the research conducted in Nepal (43%) [32]. A study done in the rural areas of Punjab, Pakistan showed 17% satisfaction with the quality of services [15]. These results show the discrepancy in the provision of services in rural and urban areas. If the clients perceive the services as of good quality, it acts as a pull factor for them, encouraging them to show up for follow-up visits, opt for institutional deliveries, and have healthier pregnancy outcomes [16]. Services that fail to meet the expectations of the users result in dissatisfaction, and consequently dropouts from the recommended revisits. The loss of follow-up then results in unwanted pregnancy outcomes, such as morbidities, mortalities, and stillbirths.

The research was carried out during the third wave of the pandemic; therefore, the results were also compared with the studies done during this period. Not much literature could be found focusing on the perceived quality of care. Rather, the focus was laid on organizational changes, fear of Covid, vaccine hesitation, etc. In a study done in Sweden during the pandemic, the perceived quality of care was rated as poor by 46.7% of expectant mothers. The study showed that one-fourth of expectant mothers complained of ineffective communication (especially regarding the information about the disease caused by the virus), and more than 20% had concerns regarding the available human resources (since they were not using PPEs as instructed by the government, thus raising questions about the quality of available services [33]). In another study which was carried out online using various social media platforms, 66% of study participants rated the quality of services as good [34]. This discrepancy in results can be due to the fact that the majority of the participants were from developed countries with better resources and telehealth systems. On the contrary, in Pakistan, such online appointment and counselling systems could not be operated due to the poor infrastructure and lack of education of the participants. In a study carried out in Pakistan, before the pandemic, only 17.5% of participants rated the overall quality of services as poor, while 61% rated it as good, and 17.5% as average [35]. During the pandemic, deterioration in perceived quality was observed as shown by the current study results. This deterioration can be attributed to organizational changes made in response to the outbreak, the reallocation of healthcare human resources leading to their shortage, fear on the part of the healthcare workers that they would catch the disease, cancellations and rescheduling of appointments, all prone to have a negative influence on the perceived quality of care. Our findings were contradictory to the findings of a study carried out in Sri Lanka, which showed satisfaction among the majority of mothers with the quality of antenatal care services during the pandemic. This disparity could be attributed to the better educational level of participants, as well as the role of public midwives in ensuring the continuity of services in the country [36]. In Tangerang City, Indonesia, expectant mothers were satisfied with the quality of care they received during pregnancy during the time of COVID, which is, again, attributed to the successfully implemented public midwife program [37]. These two studies show the strengths of public midwife programs in ensuring the continuity of care and the weakness of these programs in Pakistan. This dissatisfaction with the quality of service that was seen in our study participants can make them want to give birth at home, which is linked to poor birth outcomes, such as an increased risk of maternal death and illness, stillbirths, and neonatal death [35]. 

Information sharing and the availability of trained health professionals are crucial for gaining the trust of the patients, and were found inadequate by more than half of the participants, as they neither explained the needs for the required tests nor were they explained the test results. They were not involved in the decision-making process. A study in Sweden revealed that 26.5% of women in the developed country also felt that there was an inadequate amount of information sharing during the pandemic while they were visiting health facilities [33]. 

Anticipatory guidance, which prepares mothers for the process of delivery and labor, guides them about breastfeeding practices, and creates awareness about the significance of light exercise and nutrition, etc., was also not considered of good quality by the users. Similar findings were reported in a qualitative study done in twin cities of Pakistan during the pandemic [38]. Another qualitative study done in Pakistan during the pandemic disclosed that it was difficult to find doctors, nurses, or health centers that could provide the necessary services during that period. This highlights the challenge faced by the users during the pandemic and this uncertainty in turn leads to dropouts from scheduled appointments [10]. Pregnancy and childbirth are stressful for the mother and the whole family, as unanticipated events can happen at any stage, and if they are not sure that they will be able to reach the concerned person in time of need or that they can ask them the questions that bother them, then they lose their confidence in the system, which leads to poor utilization of health services. 

Poor educational status, low income, more than four children, and long waiting times showed associations with the perceived poor quality of healthcare services. Women who had given birth to two or more children were less likely to give birth in a medical facility than women who were giving birth for the first time, thus showing their dissatisfaction with the services provided [8]. The influence of education is significant, as women who have completed secondary or higher education had three times the likelihood of giving birth in a medical facility. Women’s satisfaction with services delivered is influenced by their educational level and unless steps are taken to include health in all policies, the desired goals cannot be achieved. Women whose households are in the top quintile of wealth have four times the likelihood of giving birth in a hospital compared to women whose households are in the bottom quintile of wealth [16]. Even though low household income should not be a barrier to accessing essential maternal health services, the findings show that those with low monthly income are dissatisfied with the service quality. A possible rationale could be that a higher household income makes it easier for women to finance travel fees and gain access to prenatal care services in private hospitals [39]. 

Long waiting times in healthcare facilities during the pandemic were a concern for women who participated in an online survey to assess the quality of healthcare services in the UK, USA, and Ireland [40]. This can be due to the fear of catching the disease while waiting in the health facility. Our results also revealed similar findings for long waiting hours in the health facility. 

### 4.1. Significance of the Study

To the best of our knowledge, this is the only study carried out during the COVID-19 pandemic that aimed at assessing the users’ perceptions of the quality of antenatal care based on their experiences at the health care facilities in the less developed areas of the country. The study highlighted the need for ensuring the continuum of care by providing quality maternal health services (even in times of disasters and pandemics) to build trust among users and increase the uptake of antenatal services in the public sector. Good quality of healthcare services, easy access to the health facility, patient-centered care, and the availability of healthcare providers, even in times of disaster, are the keys to ensuring visits to a health facility for ANC services at the scheduled time and promoting deliveries at a health institution. Implementation and monitoring of these measures will pave the way for the country to achieve SDG 3.

### 4.2. Limitations of the Study

The single-center study used a closed-ended questionnaire and did not allow women to express their views. A multicenter study that shows the strengths and weaknesses of the system, taking into account the patient’s perspective, structure, and process outcomes, may give better results. The research did not account for stakeholders’ past experiences. Results would have been more conclusive if the previous experiences were taken into consideration for the users who had used the same health facility during their previous pregnancy. 

## 5. Conclusions

The majority of pregnant mothers attending the study facility considered the provided services to be of subpar quality. Their greatest concern was healthcare experts’ availability and the time spent by service providers preparing them for pregnancy and childbirth and answering their questions. Household income, education level, number of living children, and time spent in the health facility’s waiting room greatly affect how expectant mothers perceive the quality of services. User satisfaction affects service usage. Good-quality services valued by pregnant mothers during normal times and emergencies are the only way to increase their involvement in the available health services, leading to more institutional births and improving their health and pregnancy outcomes, even in times of uncertainty, such as amidst pandemics. 

## Figures and Tables

**Table 1:** Sociodemographic characteristics of study participants.

Variables	Category	Frequency (%)
Age	18–25 years	45 (22.5)
	26–34 years	61 (30.5)
	35 and above	27 (13.5)
	Don’t know	67 (33.5)
	29.32 ^a^, 18–38 ^b^	
Participant’s education level	None	77 (38.5)
	Primary or less than primary	27 (13.5)
	Middle	23 (11.5)
	Matric	24 (12)
	Intermediate	19 (9.5)
	Bachelors and above	30 (15)
Husband’s education level	None	74 (37)
	Primary or less than primary	15 (7.5)
	Middle	28 (14)
	Matric	50 (25)
	Intermediate	17 (8.5)
	Bachelors and above	16 (8)
Place of residence	Cities	40 (20)
	Village or small town	160 (80)
Husband’s occupation	Agriculture	21 (10.5)
	Employed	16 (8)
	Labor	108 (54)
	Professional	32 (16)
	Other	23 (11.5)
Monthly household income in Rupee	Less than 10,000	4 (2)
	20,000–60,000	128 (64)
	More than 60,000	34 (17)
	10,000–19,999	34 (17)
Age when married	15–20 years	75 (37.5)
	21–25 years	95 (47.5)
	26–30 years	25 (12.5)
	Less than 15 years	5 (2.5)
Family structure	Joint family	156 (78)
	Nuclear family	44 (22)
No. of people in the household	Less than 5	40 (20)
	5–10	146 (73)
	More than 10	14 (14)
Related to husband	No	80 (40)
	Yes	120 (80)

a. Mean age (for those who knew their year of birth); b. age range (for those who knew their year of birth).

**Table 2:** Pregnancy-related characteristics of participants.

Variables	Category	Frequency (%)
Trimester of pregnancy	2nd trimester	82 (41)
	3rd trimester	99 (49.5)
	1st trimester	19 (9.5)
Number of living children	None	76 (38)
	1–2	88 (44)
	3 or more	36 (18)
	0–7 ^a^	
Number of antenatal visits	2 visits	79 (39.5)
	3 visits	8 (4)
	1 visit	113 (56.5)
	2 ^b^, 1.47 ^c^	

a. Range (number of living children); b. range (number of antenatal visits during the current study); c. mean (number of antenatal visits).

**Table 3:** Perceived hurdles in accessing antenatal healthcare services.

Variables	Category	Frequency (%)
Distance from the health facility	Yes	182 (91)
	No	18 (9)
Waiting time	Yes	166 (83)
	No	34 (17)
Participant considers pregnancy a health issue	Yes	59 (29.5)
	No	141 (70.5)
Husband’s negative attitude towards antenatal care	Yes	143 (71.5)
	No	57 (28.5)
COVID-19 restrictions	Yes	190 (95)
	No	10 (5)
Participant realizes the significance of delivery by skilled birth attendants	Yes	47 (23.5)
	No	153 (76.5)

**Table 4:** Multiple linear regression showing variables that influence the total quality of prenatal care as perceived by expectant mothers.

Predictor Variables ^b^	Coefficient Beta	Std. Error	t	*p*-Value ^a^
Intercept	2.909	0.057	51.078	0.000
Education level (none, primary or < than primary) ^b^	−0.218	0.046	−4.762	0.000
Income (<10,000 PKR) ^b^	−0.428	0.143	−2.993	0.003
Number of living children (>than 4) ^b^	−0.011	0.043	−0.263	0.033
Long waiting time	−0.1190	0.060	−1.981	0.049

a. *p*-value of less than 0.05 was considered significant; b. variables transformed in automatic linear stepwise modeling to ensure normality

## Data Availability

The data that support the findings of this study are available from the corresponding author upon reasonable request.
